# Mirroring Intentional Forgetting in a Shared-Goal Learning Situation

**DOI:** 10.1371/journal.pone.0029992

**Published:** 2012-01-05

**Authors:** Mihály Racsmány, Attila Keresztes, Péter Pajkossy, Gyula Demeter

**Affiliations:** 1 Department of Cognitive Science, Budapest University of Technology and Economics, Budapest, Hungary; 2 Institute of Psychology, University of Szeged, Szeged, Hungary; Kyushu University, Japan

## Abstract

**Background:**

Intentional forgetting refers to the surprising phenomenon that we can forget previously successfully encoded memories if we are instructed to do so. Here, we show that participants cannot only intentionally forget episodic memories but they can also mirror the “forgetting performance” of an observed model.

**Methodology/Principal Findings:**

In four experiments a participant observed a model who took part in a memory experiment. In Experiment 1 and 2 observers saw a movie about the experiment, whereas in Experiment 3 and 4 the observers and the models took part together in a real laboratory experiment. The observed memory experiment was a directed forgetting experiment where the models learned two lists of items and were instructed either to forget or to remember the first list. In Experiment 1 and 3 observers were instructed to simply observe the experiment (“simple observation” instruction). In Experiment 2 and 4, observers received instructions aimed to induce the same learning goal for the observers and the models (“observation with goal-sharing” instruction). A directed forgetting effect (the reliably lower recall of to-be-forgotten items) emerged only when models received the “observation with goal-sharing” instruction (*P*<.001 in Experiment 2, and *P*<.05 in Experiment 4), and it was absent when observers received the “simple observation” instruction (*P*>.1 in Experiment 1 and 3).

**Conclusion:**

If people observe another person with the same intention to learn, and see that this person is instructed to forget previously studied information, then they will produce the same intentional forgetting effect as the person they observed. This seems to be a an important aspect of human learning: if we can understand the goal of an observed person and this is in line with our behavioural goals then our learning performance will mirror the learning performance of the model.

## Introduction

A flexible memory needs a mechanism by which it can disregard earlier encoded information that is no longer reliable, is irrelevant or even disturbing. The experimental procedure called directed forgetting (DF) demonstrates this relevant aspect of human memory. In a typical directed forgetting experiment participants first learn a set of items, usually a list of words (henceforth: List 1), then receive an instruction either to forget or to remember these items. This paradigm is called the list method of directed forgetting and studies using this procedure demonstrated that following learning of further items (henceforth: List 2), participants can recall significantly fewer of the items designated to be forgotten compared to those that were to be remembered [1]–[4]. The experimental work of the last thirty years has revealed many attributes of the DF effect and the brain mechanisms involved in this phenomenon have also become clear [5], [6], [7]–[13].

The dominant theory of directed forgetting was framed by Bjork [1] who suggested that the forget instruction elicits a process in participants which suppresses the access of List 1 items, although this process is modulated by factors such as list segregation and recall output order. According to Bjork [1] the suppression of List 1 items serves an adaptive goal for participants to escape from proactive interference while studying List 2 items (see Racsmány and Conway [14] for an extension of this concept to episodic retrieval). This idea was supported by experimental results showing that recall performance of List 2 items is significantly higher following a forget instruction than following a remember instruction of List 1 items, although this beneficial effect of forget instruction has not been present constantly in directed forgetting experiments (see[2]). The suppression theory of directed forgetting received strong support both from neuroimaging studies and from investigations of patients suffering from brain damage or psychiatric disorders. For instance, Mecklinger, Para and Waldhauser [15] showed that successful forgetting in a directed forgetting experiment elicited a right frontal activation following the forget instruction. This brain area – and especially the right inferior frontal gyrus - is associated to inhibition of prepotent responses [15], [16] (see also [6]). Bäuml, Hanslmayr, Pastötter and Klimesch [17] showed that forget instruction induces a change in alpha oscillations which is assumed to be an active neural inhibitory filter. Furthermore, patients with lesion in the right frontal cortex and patients diagnosed with schizophrenia – known to have frontal dysfunctions [18] - were unable to produce a directed forgetting effect [11], [19].

An alternative explanation of directed forgetting was proposed by Sahakyan and Kelley [20] who suggested that the forget instruction produce a change in mental context of participants and this change serves as a key factor for later recall patterns. According to this explanation, directed forgetting is just another example of context dependent memory phenomenon. Participants in the forget group change their internal context as a response to the forget instruction, therefore they are studying List 2 items in a changed mental context and finally they try to recall List 1 and List 2 items in this new mental context. In contrast, participants who receive a remember instruction will learn both lists in the same internal context. Sahakyan and Delaney [21] suggested that only the cost of directed forgetting (the decreased List 1 recall performance in the forget group) is explained by contextual change, while other factors, such as changed learning strategy, contribute to the benefit of forget instruction (the higher recall of List 2 items in the forget group). The results of these experiments gave evidence that instructing participants to intentionally change their mental context produced the same level of forgetting of List 1 items as the ‘standard’ forget instruction (see [22]).

A fundamental difference between these two concepts of directed forgetting is the role of participant's goal in the causal explanation of the phenomenon. According to the framework of Bjork [1], suppression of the first list is a goal-related response to the forget instruction, where the goal of the participant is to learn valid and disregard invalid information. In contrast, the context change hypothesis [20] proposes that the suppression of the first list is a side effect of the instruction. The forget instruction segregates the two learning lists and creates different contexts for them, however the goal of the participant does not play a causal role in this process. It is difficult to discriminate the predictions of the two explanatory concepts in the standard directed forgetting procedure, because we should manipulate independently the goal of participants and the type of instructions they receive. However, the type of the instruction always determines the goal of the participant, thus these two factors are strongly associated in the standard DF procedure. We can discriminate these two factors, if participants are not directly instructed, but observe another person, a model, who receive a forget instruction. This way it is possible to manipulate independently the goal of the observer (congruent or incongruent with the goal of the model) and the type of instruction (forget or remember) given to the model.

Dissociating goal and instruction is also fruitful from a more general point of view. The directed forgetting procedure is a paradigmatic case of intentional learning, where a learner has to keep relevant information in an active form while has to suppress irrelevant information. From the perspective of an adaptive cognitive system we can assume that participants are able to produce an intentional suppression of successfully studied information by detecting which information is relevant and which is irrelevant for an observed model. By applying the directed forgetting procedure in an observational learning task, where the relevant information must be extracted from the interaction of the experimenter and the observed model, it is possible to get evidence for the adaptiveness of intentional forgetting.

The central question of the present study was whether or not observers were able to mirror the learning performance of an observed model who had received a forget instruction. Considering the learning process as a specific action, we aimed to investigate the role of the observer's goal in activating and suppressing memories. In research on action understanding there are many observations of an action eliciting the same brain activity pattern in motor planning areas as the actual execution of that same action [23]–[26]. Moreover, studies using various stopping paradigms have demonstrated that the observers mirrored inhibitory attention processes along with the perceived person's action [27], [28]. However, so far there has been no demonstration of mirroring explicit goal-related memory access.

According to our hypothesis, observers can mirror the intentional forgetting performance of an observed model, but only if they share the same goal in the learning situation. If the observers' goal is simply to observe the behaviour of the observed model, they will not mirror intentional forgetting; therefore, they will remember the to-be-forgotten information. We assume that a forget instruction elicits suppression of earlier encoded information only if this instruction targets goal relevant information for the observer.

We developed a modified version of the DF procedure aimed at investigating whether or not participants are able to simulate the intentional forgetting performance of a model. In this experimental procedure, called observational directed forgetting (oDF), participants (the observers) observe another person (the model) taking part in a directed forgetting experiment.

## Methods

We have obtained ethics approval for our study from the ethics committee of the Budapest University of Technology and Economics, Hungary, all participants gave written consent.

### Experiment 1 & 2

In two consecutive experiments, a total of 200 native Hungarian speakers were recruited from the Budapest University of Technology and Economics student population. They received course credits for their participation. One hundred participants (45 males and 55 females) took part in each experiment, their ages varied between 19 and 26 years.

In both experiments, participants (referred to as observers throughout the article) watched a movie of a directed forgetting experiment. In this movie, a model learnt a list of words (List 1), then received a midlist instruction (forget or remember), then learnt another list of words (List 2). In both experiments, observers were randomly assigned to either the forget or the remember group.

The two experiments differed only in the instruction given to the observers prior to watching the movie. In Experiment 1, they were told simply to observe everything they saw in order to remember it later on (“simple observation”), whereas in Experiment 2 observers were told to observe everything they saw in order to remember what the model in the movie had to remember (“observation with goal sharing”).

In the movie presented to the observers, a male model sat in front of a computer screen and was told by an experimenter that he would be presented with a list of words and that his task was to learn all of the words for a later memory test. Each word was displayed for 2 s with a 2-s inter-item interval. When filming the movies we used two experimental learning lists (List A and List B) consisting of 12 words of moderate to high frequency. Half of the observers saw a version of the movie in which List A served as List 1, and List B served as List 2, while the other half of the observers saw a version in which List A served as List 2 and List B as List 1. After List 1 had been presented on the screen the experimenter gave either a “forget” or a “remember” instruction. In the “forget” condition, the model in the movie received the instruction that the words presented up until this point were only presented by mistake, and the experimenter asked the model to try to forget these words in order to properly carry out the learning of the following words. Following the forget instruction the model was presented with a second list of 12 words. In the “remember” condition, the experimenter in the movie gave a remember instruction following List 1; that is, he asked the model to remember the words presented up until that point and to try to learn the words in the second list as well. Following the presentation of List 2, the experimenter thanked the model for their contribution.

Following the presentation of the movie, observers took part in a distractor task in which they solved simple arithmetic tasks for 10 min. Then they were asked to recall all the words that had been presented to the model in the movie. All observers were first asked to recall List 1, and then List 2 words, in order to avoid a possible output interference of List 2 words in the forget condition.

### Experiment 3 & 4

In two further experiments, a total of 208 native Hungarian speakers were recruited from the Budapest University of Technology and Economics student population. One hundred-twenty participants (43 males and 77 females) took part in Experiment 3, and eighty-eight participants (39 males and 49 females) took part in Experiment 4. Their ages varied between 19 and 28 years. Data of four participants (two models and one observer) was excluded from the analysis of Experiment 3, and data of three participants (two models and one observer) was excluded from the analysis of Experiment 4, because they figured out the goal of the experiment, as it was revealed by the debriefing.

The two experiments followed the same logic as Experiment 1 and 2 with the only exception that this time the observed model was a real participant, not only an actor in a movie.

Two participants (one model and one observer) took part in the experiment at the same time. Each participant pair (observer and model) was randomly assigned to either the remember or the forget group and each member of the pair was randomly assigned to be the observer or the model in the experiment. First, the observers were informed that they would take part in a memory experiment as an observer where a model would learn lists of words for a later recall. The observer was also informed that the aim of their participation is to warm up for a later memory experiment. Similarly to Experiment 1, in Experiment 3 observers received a “simple observation” instruction; that is, their task was to watch carefully and observe everything they saw, because later they would have to remember it. Similar to Experiment 2, in Experiment 4 observers received an “observation with goal sharing” instruction; that is, their task was to watch carefully and observe everything they saw, but crucially they were also informed that at the final recall test there would be a possibility to help the model if she/he asks for it.

The model and the observer sat close to each other in front of a computer screen, in a distance from the screen so that both of them could easily read the presented stimuli. Each word was displayed for 2 s with a 2-s inter-item interval. The experimenter gave instructions only to the model, who were informed that they would be presented with a list of words and were to learn all of the words for a later memory test. After the first list of words had been presented on the screen the experimenter gave either a “forget” or a “remember” instruction to the model. In the “forget” condition the models received the instruction that the words presented up until that point were only presented by mistake, and the experimenter asked them to try to forget those in order to properly carry out the learning of subsequent words. After the forget instruction the models were presented with a second list of words. In the “remember” condition the experimenter gave a remember instruction following List 1, asking the models to remember the words presented up until that point and to try to learn the words on the second list as well.

After the presentation of List 2, both the models and the observers took part in a distractor task, solving simple arithmetical problems for 10 minutes. Then they were asked to recall all the words that had been presented to the model in the movie. All observers were first asked to recall List 1, and then List 2 words, in order to avoid a possible output interference of List 2 words in the forget condition.

## Results

In all four experiments the same mixed ANOVA was carried out with instruction (Forget/Remember) as between subject variable and list (List 1/List 2) as within subject variable. In Experiment 3 and 4, recall data of models and observers were analysed separately, and when discussing these results, we report data for models first, and data for observers second.

### Experiment 1

**Figure 1:**
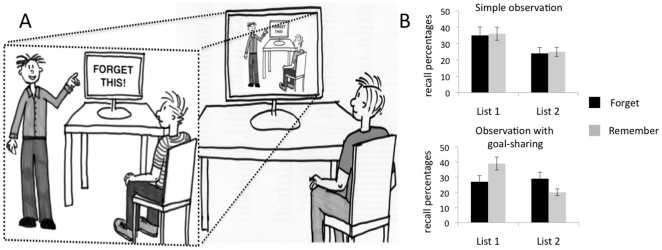
Experimental set-up and results of Experiment 1 and 2. (A) In both experiments the observers sat in front of a computer screen on which they saw a movie of a directed forgetting experiment. In this movie, a model was instructed to learn a list of words shown on a computer screen, and was then shown a second list that was also to be learnt. Immediately before being presented with the second list to learn, the model in this movie received a midlist instruction. Half of the observers saw a movie where the model was instructed to forget the list that they had seen before and to learn the second list (this is the forget condition shown here). The other half of the observers saw a movie where the model was instructed to remember the second list as well (the remember condition). In experiment 1 (upper part of panel B), the observers were simply told to observe the movie in order to remember as many details as possible (simple observation). Here, we found no directed forgetting effect: after watching the movie the observers recalled a similar number of words in the two conditions, *P*>.1. In experiment 2 (lower part of panel B), the observers were told to observe the movie in order to remember everything that the model in the movie had to remember (observation with goal-sharing). Here, we found a significant directed forgetting effect: after watching the movie the observers in the forget condition recalled significantly fewer words from the first list and recalled significantly more words from the second list than the observers in the remember condition, *P*<.001.

We found a significant main effect of list, *F*(1,98) = 13.15, *P*<.001, but no significant interaction between list and instruction, *F*(1,98) = 0.02, ns. Independent t-tests showed that, on average, observers in the forget group and the remember group recalled the same proportion of List 1 words, *t*(99) =  −0.67, ns., and the same proportion of List 2 words, *t*(99) =  −0.66, ns. This supports our hypothesis that observers with an attitude of merely observing a learning action of a model will not produce the same memory performance as the observed model; therefore, they will not produce an intentional forgetting of List 1 in the forget condition (see Figure 1, upper part of panel B).

### Experiment 2

The same ANOVA as in Experiment 1 yielded a significant main effect of list, *F*(1, 98) = 20.08, *P*<.001, and more importantly, a significant interaction between list and instruction, *F*(1,98) = 17.4, *P*<.001. Independent t-tests revealed that observers in the forget group recalled fewer List 1 words, *t*(99) = 2.19, *P*<.05, *r* = .22, but more List 2 words, *t*(99) = 2.83, *P*<.01, *r* = .27, than observers in the remember group. This recall pattern shows that our manipulation was successful in inducing a directed forgetting effect. (see Figure 1, lower part of panel B).

### Experiment 3

#### Models

**Figure 2:**
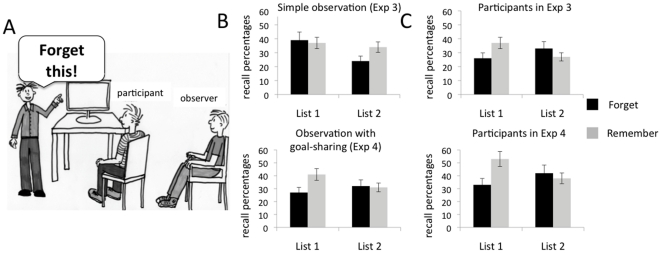
Experimental set-up and results of Experiment 3 and 4. (A) In both experiments participants sat in front of a computer screen and participated in a directed forgetting experiment (we refer to these participants as models and their results are shown in panel C). They were instructed to learn a list of words (List 1) shown on the screen. Immediately after being presented with List 1, the models received a midlist instruction. Half of the models was instructed to forget the list (List 1) they had seen before, and learn the second list (List 2). This is the forget condition shown here. The other half of the models was instructed to remember List 2 as well (remember condition). Models (panel C) in both Experiments showed directed forgetting. Each model was observed by another participant (we refer to these participants as observers and their result are shown in panel B). In experiment 3, observers (upper part of panel B) were told simply to observe the experiment in order to remember as many details as possible (simple observation). Here, we found no directed forgetting effect: after watching the experiment, observers in the forget and remember condition recalled a similar number of List 1 words, and observers in the remember condition recalled more words from List 2 than observers in the forget condition. In experiment 4, observers (lower part of panel B) were told to observe the experiment in order to be able to help the model in the experiment (observation with goal-sharing). Here, we found a significant directed forgetting effect: after watching the experiment, observers in the forget condition recalled significantly less words from List 1 than observers in the remember condition.

The list X instruction interaction was significant, *F*(1,58) = 10.56, *P*<.005. Independent t-tests revealed that models in the forget group recalled fewer List 1 words, *t*(58) =  −2.67, *P*<.01, *r* = .33, but more List 2 words, *t*(58) = 1.29, ns., than models in the remember group. Although this latter effect, the benefit of directed forgetting instruction, was not significant, our manipulation was successful in inducing a directed forgetting pattern among models (see Figure 2, upper part of panel C).

#### Observers

Observers showed a different pattern compared to the models they had observed. Their recall data showed no significant list X instruction, *F*(1,54) = 2.54, *P* = .117. Also, independent t-tests revealed that observers in the forget group recalled a similar proportion of List 1 words, *t*(54) = 0.43, ns., and a lower proportion of List 2 words, *t*(54) = 1.69, ns., compared to observers in the remember group. In brief, in this group we found no directed forgetting effect (see Figure 2, upper part of panel B).

### Experiment 4

#### Models

The list X instruction interaction was significant, *F*(1,40) = 12.34, *P*<.001. Independent t-tests revealed that models in the forget group recalled fewer List 1 words, *t*(40) =  −3.47, *P*<.001, r = .48, but more List 2 words *t*(40) = 0.51, ns., than models in the remember group. Again, as for models in Experiment 3, although the benefit of directed forgetting instruction was not significant, our manipulation was successful in inducing a directed forgetting pattern among participants (see Figure 2, lower part of panel C).

#### Observers

In contrast to Experiment 3, observers in Experiment 4 showed a similar pattern as the models they had observed. Their recall data showed significant list X instruction, *F*(1,41) = 4.24, *P*<.05. Also, independent t-tests revealed that observers in the forget group recalled fewer List 1 words, *t*(41) =  −2.36, *P*<.05, *r* = .35, and a similar proportion of List 2 words, *t*(41) = .12, ns., compared to observers in the remember group. In brief, although we found no benefit of the directed forgetting instruction for the forget group, the observers showed a clear directed forgetting effect (see Figure 2, lower part of panel B).

## Discussion

In Experiment 1 and 2 we demonstrated that observers mirror the effect of the forget instruction given to an observed model. This mirroring only occurred when the instruction given to the observers induced shared goal representations.

Although Experiment 1 and 2 gave evidence that suppression of the to-be-forgotten items is modulated by the observer's goal, the applied instruction and the specific way of item presentation raised a series of question with respect to the above interpretation of our results. Did the instruction to remember everything that the model had to remember induce any empathy/goal sharing with the model, or did the observers simply interpret the instruction given to the model as an instruction given to them? Another problem in our interpretation might be that the model did not suppress memories (models were actors in a movie). Therefore we cannot infer that the forgetting effect produced by the observers is truly a mirrored effect.

To clarify these questions we changed both the learning situation and the goal-sharing instruction in two following experiments. In Experiment 3 and 4, instead of watching a movie about an experiment, observers observed a directed forgetting experiment in a real-life setting, with real experimental participants (models). In order to induce empathy/goal sharing of observers with the models, we changed the “observation with goal-sharing” instruction of Experiment 2 in a way to stress the shared goal of the two persons. Therefore, the observers were told that they could help the model at the final recall. We reasoned that this instruction not only induces shared goal-representations, but also rules out the possibility that observers simply interpret the instruction given to the model as an instruction they (the observers) should follow. Besides this, the real-life setting, used in Experiment 3 and 4, allowed us to match the recall pattern of observers to the recall pattern of real participants.

The results of Experiment 3 and 4 replicated the results of Experiment 1 and 2. That is, observers mirrored the effect of the forget instruction given to the observed model, but only when the instruction given to the observers induced shared goal representations.

In sum, we demonstrated that directed forgetting effect in the observer was only present if the goal to encode specific memories was the same or similar for the observer and the model. In four experiments we gave evidence that observers suppressed List 1 items if they observed a model who was instructed to forget these items. However, this effect was modulated by the instruction type given to the observers. Observers only produced the directed forgetting effect if they were instructed to share the goal of the model. This means that if the observer's goal is to acquire the same information as the model, then any environmental manipulation of the model's behaviour will influence the accessibility of the observer's memories. It is important to note that goal sharing was manipulated in two fundamentally different ways in Experiment 2 and Experiment 4. In Experiment 2 observers watched a movie about the experiment, they had no contact with the models, and because of this one could argue that observers may have not felt empathy for the models or shared the model's goal. More importantly, as the observer were instructed to remember everything that the model in the movie had to remember, this may have forced them to instruct themselves the same way as the experimenter instructed the model. However, in Experiment 4 observers took part in the same experiment as the model: they sat next to them and they followed their behaviour from close distance. This experimental design should have induced more empathy in the observers for the model. Moreover, the instruction also differed in Experiment 4. Observers were instructed that they might have the chance to help the model at the final test. This instruction probably led the observer to share the goal of the model. Although there are major differences in the observer's instructions in Experiment 2 and 4, the two experiments produced exactly the same pattern of results. This supports the conclusion that shared goal of observers and models was the critical factor in producing this observational directed forgetting effect.

A further contribution of Experiment 3 and 4 compared to Experiment 1 and 2 is that the memory performance of the model is known. The direct comparison of observers' and models' performance gave further evidence that observers mirrored the memory performance of the model in Experiment 4, while their performance was different from that of the model in Experiment 3.

In a narrower interpretation, our results provide relevant evidence for theoretical accounts of directed forgetting. The concept of retrieval inhibition [1] states that the forget instruction, together with further learning of List 2 triggers an inhibitory process in order to attenuate the interference of to-be-forgotten items with to-be-remembered items. Inhibitory processes serve an adaptive role to enhance the accessibility of reliable items and suppress all unimportant and disturbing information. In contrast, the context change hypothesis [20] proposed an account without inhibition by suggesting that participants in the forget group will create a larger than normal change in internal contextual elements, and will treat the two study lists as separate events because of the forget instruction. As a consequence, participants in the forget group will encode List 1 words in a different context than List 2 words, and there will be a contextual mismatch between List 1 and final recall. According to this concept the forget instruction plays no specific role in the directed forgetting phenomenon, and it is replaceable with any other manipulation causing a similar contextual change between the two study lists.

In our opinion the results of the present study fit better to the concept of retrieval inhibition than to the context change hypothesis. The forget instruction will carry the future importance of studied information only if it targets goal-relevant aspects of the previous event. In other words, the forget instruction will trigger inhibitory processes for to-be-forgotten information, because it informs the participant that these items are no longer relevant from the perspective of the present goal of the learner, that is, the successful recall of the studied items. An observer without the goal to recall all relevant information from the point of view of the model will not use the information of the forget instruction.

It is unclear, how the context change hypothesis could explain the present results in a parsimonious way. To explain the recall performance of the observers with this concept we should assume that observers without shared goal with the models did not create a new internal context for the second list as a response to the forget instruction. Contrary, they have changed their mental context if their goal was in accord with the model. Following the logic of this account we should assume that the “observation with goal sharing” instruction increased the encoding of contextual elements compared to the “simple observation” instruction. One problem with this explanation is that there has been no evidence for such an association between goal-directed learning and internal context encoding. Another, and more evident, problem is that enhanced contextual encoding should have lead to a higher average recall rate among observers instructed with “observation with goal sharing” instruction. This is certainly not the case.

In sum, these results underlie the general assumption that activation and suppression of episodic memory representations is based on goal-related action plans [29]. It is important to note that it has been widely documented that the suppression effect in the directed forgetting procedure lowers the accessibility, but not the availability of to-be-forgotten memories, meaning that these memory items remain intact but become inaccessible by episodic retrieval cues [2], [14], [30]. Our results support the assumption that suppression of episodic memories is not automatically generated by environmental cues but depends on the goals of the person who encodes and retrieves them [29]. In a broader interpretation, these results gave evidence that observers can mirror the suppression memory effect of the model if they take the model's action goals. The central question of action mirroring is whether the mechanism is a direct match between the perception of the model's action and the observer's motor system [26] or whether it is generated from goal interpretation via top-down processes [31]. Our results suggest that the mirroring of intentional forgetting takes place in the latter form. When the observer shares the model's goal, they will encode items that are relevant to the model and then they will manipulate the accessibility of their own memories according to what seems to be relevant to the model in a learning action. The exact nature of this process – whether it is an action simulation or an end state emulation by different means – is presently unclear, but our results point to a relevant aspect of social learning. Human learners manipulate the activation level of their own memory according to the specific goal of the observation, and if this goal matches the goal of the observed model than the observer will mirror the learning performance of the model.
